# Bibliographical Notices

**Published:** 1848-07

**Authors:** 


### EDITORIAL DEPARTMENT.

### BIBLIOGRAPHICAL NOTICES.

Several new publications have lately been received. Of some of these our notice at this time is intended merely as a matter coming under the head of medical intelligence, not precluding thereby, critical or analytical reviewal hereafter, should it be deemed advisable.

*Lectures on the Theory and Practice of Physic.* By JOHN BELL, M. D., etc., and WILLIAM STOKES, M. D., etc. Eourth Edition, Revised and Enlarged. 2 volumes. Philadelphia: Ed. Barrington & Geo. D. Haswell. 1848. 8 Vo. pp. 976.

It is unnecessary to speak of the character of this work, as it is one with which the medical profession of this country are sufficiently acquainted. The present is the fourth edition, which has been carefully revised, and enlarged by the addition of new and useful matter. Since the portion contributed by Dr. Bell already considerably exceeds that taken from Dr. Stokes, and is increasing with each new edition, we hope that, by and by, the work will become exclusively a work by Dr. Bell. We make this remark without wishing to be understood to depreciate the valuable lectures by Dr. Stokes, for this would show as little discretion as discrimination. But, we confess, such partnerships are not to our taste. Dr. Bell has certainly demonstrated his ability to prepare a work on practice which shall prove satisfactory to the profession: let us then have ***Bell's practice,*** as an American work, leaving (to use a homely phrase, which we trust the fastidious reader will excuse) "every tub to stand on its own bottom."

---

*On Poisons, in relation to Medical Jurisprudence and Medicine.* By ALFRED S. TAYLOR, F. R. S. etc. Edited, with notes and additions, by R. EGLESFELD GRIFFITH, M. D. Philadelphia: Lea and Blanchard. 1848. 8 vo. pp. 687.

The object of this work is to present a more extended view of the subject of Toxicology, than is contained in works on Medical Jurisprudence. Dr. Taylor is well known as author of a valuable treatise on the latter subject. The present volume is published in a form corresponding to that work. In the words of the American Editor (whose name is a sufficient guaranty of the value of the work) "it is an elaborate epitome of all that is known on the subject of Poisons, and is amply illustrated with cases, so as to exemplify the relative value attached by juries to the various symptoms induced by the respective articles; and also of the modes of detecting these articles, as given by the best authorities."

It must be an useful addition to the library of the Medical Practitioner.

---

*Principles of Physics and Meteorology.* By J. Muller, Professor of Physics at the University of Freiburg. First American Edition. Revised and illustrated with 538 engravings on wood, and two colored plates. Philadelphia: Lea & Blanchard. 1848. 8 vo. pp. 635.

By the Publishers' advertisement, we learn that this Treatise is one of a series of valuable works now being issued in London; and that from its thorough character and extended range, it is much esteemed in England.

With reference to the subjects which it embraces, we quote the following from the preface by the London translator:—"The subjects of which this volume treats, are very numerous—more numerous, in fact, than at first sight it would seem possible to embrace in so small a compass. The author has, however, by a system of most judicious selection and condensation, been enabled to introduce all the most important facts and theories relating to Statics, Hydrostatics, Dynamics, Hydronamics, Pneumatics, the laws of the Motion of Waves in general, Sound, the Theory of Musical Notes, the Voice and Hearing, Geometrical and Physical Optics, Magnetism, Electricity and Galvanism in all their subdivisions, Heat, and Meteorology, within the space of a middle sized volume."

The mechanical execution of this work deserves mention, albeit it is from the press of Lea & Blanchard, whose publications, in this respect, are pre-eminently excellent. It is a beautiful book, calculated to adorn, as well as enrich the Physician's study.

---

*Elements of Natural Philosophy; being an experimental introduction to the study of the Physical Sciences.* By Golding Bird, M. D., F. R. S., F. L. S. etc. etc. With three hundred and seventy-two illustrations. From the revised and enlarged third London edition. Philadelphia: Lea & Blanchard. 1848. 12 mo. pp. 402.

A short extract from the Author's preface will explain the object and scope of the above work:—"The following manual is chiefly intended as a text book for the student, whilst attending lectures on Physics, or as preparatory to his entering upon the study of larger and more elaborate works. With this view it has been written; and as the great difficulty experienced in executing this task has arisen from the necessity of knowing, not what to insert, but what to omit, whenever a doubt has arisen on this point, it has been determined by a reference to the amount of knowledge required of the student, by the different English and Scottish medical boards."

The importance of the study of Physical science, as an element of medical education, is too obvious to require comment. If we mistake not, an elementary hand book, designed to introduce the medical pupil to this study, is a desideratum. In the high attainments of Dr. Bird, and his eminence as a teacher, we have an assurance of his ability to prepare a work which shall correspond with the present state of physical science, and be adapted to the particular wants of the Medical student. From a cursory examination of this publication we have no doubt that it is such a work as we should expect from the quarter whence it emanates, and we do not hesitate to recommend it to those for whose benefit it has been prepared.

---

*On disorders of the Cerebral Circulation and of the connection between Affections of the Brain and Diseases of the Heart.* Bw George Burrows, M. D., etc. With colored plates. Philadelphia: Lea & Blanchard. 1848. 8 vo. pp. 216.

We are glad to see a re-publication of this little treatise, having, previously, known somewhat of its character and contents, by quotations in some of the British periodicals. It will serve to correct some errors in pathology, which, from the sanction of high authority, had become somewhat current, and which were calculated to lead to serious errors of practice. It is gratuitous to remind our readers of the experiments of Dr. Kellie, which appeared to show that after animals were bled to death, the brain, instead of being found exsanguine, like the other parts of the body, frequently presented marked turgidity of the vessels. Upon these experiments, Dr. Abercrombie and others, based the hypothesis that the brain, being enclosed within a complete bony sphere removing it from atmospheric pressure, and the cerebral mass being incompressible by any amount of force which the heart and other forces carrying on the circulation can exert, the encephalic vessels must necessarily, not only be always full of blood, but that the quantity of blood within the cranium can neither undergo increase or diminution. The pathological and practical consequences of this hypothesis are at once apparent. It would follow, for example, that pathologists have been hitherto mistaken in attaching importance to an afflux of blood to the brain, as a morbid condition important in itself, and still more important in its consequences. Dr Clutterbuck, indeed, in his article on apoplexy in the Cyclopedia of Practical Medicine, states distinctly that the phrase "determination of blood to the head," a phrase so often in the mouths of practitioners, is without any meaning. It would also follow that the commonly received explanation of syncope, is erroneous; and, what is of immense consequence, vascular depletion, according to this view, becomes, comparatively, an inoperative method of treating those cerebral affections in which it has heretofore been esteemed to take precedence of all other therapeutical measures. That such an hypothesis should ever have been maintained by so accurate an observer, and sound thinker as Abercrombie, is truly surprising, and serves to illustrate the danger, from which the greatest minds are not exempt, of being betrayed into hasty conclusions which are at variance with the plainest and most familiar facts. Putting aside the fallacy of reasoning, and the errors of physical science, which such an hypothesis involves, the evidence afforded by a few autopsies of patients dead with different diseases, is unequivocally opposed to the doctrine referred to, and must satisfy any unbiased observer, that error must be somewhere involved in Dr. Kellie's experiments. Dr. Burrows has repeated these experiments several times, with different animals, and arrives at results directly opposite to those just mentioned. These experiments are detailed in the work before us.

They consist in destroying animals by strangulation, and by bleeding *ad mortem*, and in suspending the bodies directly after death, in some instances by the heels, and in others by the ears, etc. Examination of the brain, under these different circumstances, demonstrated that its vascularity was thereby differently affected in a notable degree. Dr. Burrows also shows by an analysis of Dr. Kellie's experiments, that they do not justify the conclusions which have been deduced from them.

That the circulation within the cranium, however, is subject to peculiarities which do not apply to any other part of the body, is an important fact which is not to be overlooked. It is a fact involved in some pathological conditions, which can only be understood, and properly managed, by reference to it. That the brain is removed from atmospheric pressure, except in so far as the latter is exerted upon it indirectly through the vascular system, by acting upon the surface of the body generally, is probably true; and in consequence of this physical fact, the encephalic vessels must necessarily be affected by circumstances which influence the forces carrying on the circulation generally, less readily, and in a less degree, than other portions of the organism. Hence, upon the principle of the syphon, when blood is not transmitted in sufficient quantities through the arteries *to* the brain, it is, by way of compensation, retarded in its passage *from* the brain through the veins. This explains the occurrence of cerebral congestion, in other words venous stagnation, in anæmia, and whenever the action of the heart, from any cause, is weakened so as not to carry on the circulation with normal vigor. It is well known that congestion of the brain, and even apoplexy, may be produced by causes just alluded to. This important pathological principle is involved in the development of coma in various diseases, such as cholera infantum, fevers, &c. The practitioner cannot with safety to his patient loose sight of this principle. This, however, is very far from admitting the doctrine that the quantity of blood within the head admits of neither increase nor diminution. While Dr. Burrows satisfactorily refutes this doctrine, he does not appear to us to attach sufficient practical importance to the physical considerations just adverted to. Dr. B. lays great stress on the effect of vascular pressure exerted by the blood, and by varying conditions in quantity of the cerebro spinal fluid. We are disposed to differ from the author as respects the latter point. Many of the pathological results which he attributes to modifications of pressure, seem to us rather due directly to stagnation of the circulation in the encephalic vessels, in other words, to the presence of unoxygenated blood, in an undue relative proportion.

The subjects to which the foregoing remarks have reference, are treated of in the first section of the work. The second section treats of the "*connection between affections of the brain and diseases of the heart.*" The knowledge of the existence of such a connection is one of the developments of Modern Medicine. Until quite recently it was entirely overlooked. The observations of Bouillaud, Hope, Latham, and others, havh established the very frequent dependence of the former upon the latter. Dr. Burrows has collected, from various authors, 132 cases of apoplexy, or sudden hemiplegia, with reference to the co-existence of cardiac disease. Of these, 84 presented disease of heart. The inference is "that in any given number of cases, of apoplexy and sudden hemiplegia, no less than three-fifths will present unequivocal signs of cardiac disease; either hypertrophy, dilatation, valvular disease, or some combination of these lesions."

The affections of heart most frequently found in this connection are hypertrophy and valvular disease combined, although, not unfrequently, simple hypertrophy, and valvular disease without hypertrophy, are thus associated.

Dr. B. adduces statistics to determine the periods of life most prone to apoplexy and hemiplegia. He infers from data which he presents, first, that "the relative frequency of apoplexy steadily increases from 20 to 80 years of age," and, second, "that the actual number of apoplectic cases increases in each successive decennial period upwards from 20 to 70 years, of age, while the number living gradually diminish."

After some practical observations on the treatment of apoplexy, &c., the author passes to notice some, "*affections of the brain and spinal cord depending on acute diseases of the heart.*" It is a curious and important fact that cardiac inflammation sometimes occasions phenomena referrible to the brain on nervous system, simulating various affections, which render the disease of heart liable to be overlooked by the practitioner. The author cites cases in which apparent inflammation of the brain, nervous irritation, insanity, chorea, tetanus, etc., occurred in connection with pericarditis, the local symptoms of the latter being so merged in the former, that the true disease passed undetected. These cases are highly interesting, and enforce valuable lessons of diagnosis.

In concluding this notice, it is superfluous to add, that Dr. Burrows' work in our opinion, contains much useful matter in a small compass, and that we cordially commend it to the medical reader.

---

*Practical Observations on Certain Diseases of the Chest, and on the Principles of Auscultation.* By Peyton Blakiston, F. R. S., etc., etc., Philadelphia: Lea & Blanchard. 1848. 8vo. pp. 384.

The first 80 pages of this work, are devoted to the theory and practice of Auscultation. About the same number of pages are appropriated to the consideration of Thoracic Aneurism. Chronic Heart diseases are considered next in order, occupying about the same space. Next, Chronic Pleurisy, 28 pages. Next, Plastic Pneumonia, 21 pages; and, lastly, Phthisis Pulmonalis, 55 pages. It will be perceived, as the title page also intimates, that the work does not profess to be a comprehensive treatise on Pulmonary Diseases. The affections which are treated of, are illustrated by numerous cases that have occurred under the author's own observation. Indeed the work consists, chiefly, of facts illustrating the history, diagnosis, pathology, etc., of the particular affections treated of, derived from the author's experience, which appears to have been quite extensive. We have perused the work with pleasure and profit, and consider it a valuable contribution to the literature of Pulmonary diseases.

*The Young Stethoscopist; or the Student's Aid to Auscultation.* By HENRY J. BOWDITCH, M. D., one of the Physicians to the Massachusetts General Hospital. Second Edition. New York: Samuel S. & William Wood, 261 Pearl St. 12mo. pp. 303.

The first edition of this work appeared in 1846, and was noticed at some length in this Journal, (vol. 2d.) In the opinion which we then expressed of the character of the work, we have been sustained by the favor with which it has been received by the Profession, and the call for a second edition. The author was attacked in one of the last numbers of the British and Foreign Medical Review, by an anonymous reviewer, on the score of having appropriated from the works of others on the same subject—a charge as ridiculous, as unmanly. A singular occasion for reproach, truly, that the author of an elementary treatise on Auscultation has gathered important principles from various sources!

A writer of this day, may fail to add newly developed principles to those which the science of physical exploration already embraces, or to disprove principles which have become firmly established, without affording just occasion for complaint; and if he succeed in so adapting present knowledge to the mind of the student, that it will be more clearly appreciated in its important practical relations, he certainly is entitled to the credit of having done the 'state some service.' We much doubt if the late editor of the British and Foreign Review, (who, by the by, was British Editor of Laennec's treatise,) can designate a British work which is less obnoxious to the charge of want of originality, than that by Dr. Bowditch. We fancy he would find it equally difficult to select a British work, similar in its scope, which is so well adapted to fulfil the objects for which it was prepared.

We repeat here, what was said in our former notice, that we should be better pleased with the work if it embraced the complete diagnosis of diseases of the chest, associating symptoms and signs, which, in our opinion should not be dissevered in books, more than in practice. We would also again express the hope that Dr. B. may be induced hereafter to extend the scope of the work, and thereby increase the obligation under which he has already placed the profession.

---

*Notes on the Theory of Human Existence:* comprising remarks on *Vitality*, the *Mind*, and incidentally the *Soul*. The whole being an exposition of the *Nature, Powers and Destiny of Man*. By THOMAS L. WRIGHT, M. D. Publishing Agents, Robinson & Sons, Cincinnati, Ohio: 1848.

A 16mo. pamphlet containing 37 pages, for which we are indebted to the author, now lies on our table, bearing the above promising title. As the author claims so little space, in this book-making age, for so extensive a theme, we were induced to climb the ladder of our hope anew, accepting his promised aid in solving the riddle of our being. Enlisting readily in the enterprise, and, passing the Preface, at once we come to the Introductory Chapter, which treats, in the first place, of the Elements of Human existence; therein we are told that

"Physical Life, a Mind, and a Soul, embrace all that is essential to the idea of humanity. * * * The progress of science reveals to man how narrow are the boundaries to his knowledge. It displays new and broad fields of inquiry concerning the intimate nature of Agencies; silent and *mysterious* in their operation, producing results wonderful and magnificent, though common and familiar to all. In pursuing such inquiry, however, the mind speedily encounters obstacles which it *cannot surmount.* To comprehend the intangible, imperceptible agencies in operation about him; to know the Mysterious, the Infinite, the Eternal, is the ardent, constant desire of man. To accomplish it he spends wealth, and toil and years; he puts in requisition the mightiest efforts of his mind; he studies, reasons, ponders. Imagination, taxed to her utmost, wings her highest, proudest flights—but Time, and Wealth, and Intellect fail, and the delusive images of imagination and fancy, satisfy not the longings of the hungry soul. While the chains of mortality bind the mind to a corruptible, changing body, *man never can attain to such knowledge.* Fetters which he cannot shake off, weigh down his efforts, a *dim mist obscures his vision, yet rolling, changing, varying always.* Sometimes, almost, he thinks he can see the brightness of day; he can almost strain his fettered arms to the object of his desires; but—no! he is *blind* still, *bound* still!"

Having read the Title, a thermometer at fever heat, we are suddenly plunged into this Introduction, a zero, below the freezing point; and, having escaped the Russian bath, we were not a little staggered by the reaction to our hopes which had been set up. But we have persevered—read the pamphlet, been gratified, and passed more gradually through the stages of our first plunge, and finally came to this conclusion:—Our author is skilled in the trade of book-making, in so far as he commenced with his Title, wrought out his Text, and thereafter wrote the Conclusion, which by transposition or design, has found place as an Introductory chapter. And, though a little staggered at first, we finally, while considering the theme, in connexion with those who have gone before, yield to his statement that "history teaches that the Intellect is the same forever." "Man does not live for posterity" and that "consequently man's *destiny* cannot relate to others but himself.—He lives for himself alone," *p.*9. Hence we hear our author—

"With regard to the connexion of mind itself, and its instrument, nothing is known. * * * Viewing the subject subordinately in its instrumentalities, it would seem as though the *whole world* was created to furnish the conditions of the existence of the mind in the human body, through the instrumentality of the nervous system, that *all things* exist for the perfection and complete development of the nervous system in man." *p*30.

"That mind has not increased the smallest iota in strength and capacity." in some quarters, we feel assured, for, before this, man was heard to exclaim,

> " See *all things* for my use,"
> " 'See man for mine ' replied the pampered goose."

Willingly we stand aside and leave to these pre-occupants the field for debate.

Prof. MUSSEY, now of Cincinnati, was wont to relate that a college friend of his chose for a graduating theme " On the adaptedness and fitness of things." What *eclat* he obtained, in its treatment, we are not informed, but the title is taking and comprehensive, and *much* might be said upon it. That our author has chosen equally as wide a range, cannot be questioned—that he is a searcher for truth, and no pseudo-lover of it—that he has collected physiological truths which even in their present connexion will repay reading, we venture to affirm, while at the same time we cordially re-affirm his statements that—

" The organized body, and some of the characteristic features of the mind have been treated of. Concerning the Soul, there is great indefiniteness in the idea of its precise characteristics. Consequently a lengthy attempt at an exposition would only add to the mystery. As soon as what can be known, with reasonableness or certainty, is stated, on any obscure topic, it is then becoming to cease, for farther remark cannot increase the light; therefore if it do any thing, it must mystify, what is actually known."

Two pages and a half of the pamphlet, judiciously, " concern the Soul," though remotely, and the author adds but little therefore to that perplexing and perplexed mystery. W. T.

